# From Images to Loci: Applying 3D Deep Learning to Enable Multivariate and Multitemporal Digital Phenotyping and Mapping the Genetics Underlying Nitrogen Use Efficiency in Wheat

**DOI:** 10.34133/plantphenomics.0270

**Published:** 2024-12-19

**Authors:** Jiawei Chen, Qing Li, Dong Jiang

**Affiliations:** Plant Phenomics Research Centre, Academy for Advanced Interdisciplinary Studies, Collaborative Innovation Centre for Modern Crop Production, Co-sponsored by Province and Ministry, College of Agriculture, State Key Laboratory of Crop Genetics & Germplasm Enhancement and Utilization, Nanjing Agricultural University, Nanjing 210095, China.

## Abstract

The selection and promotion of high-yielding and nitrogen-efficient wheat varieties can reduce nitrogen fertilizer application while ensuring wheat yield and quality and contribute to the sustainable development of agriculture; thus, the mining and localization of nitrogen use efficiency (NUE) genes is particularly important, but the localization of NUE genes requires a large amount of phenotypic data support. In view of this, we propose the use of low-altitude aerial photography to acquire field images at a large scale, generate 3-dimensional (3D) point clouds and multispectral images of wheat plots, propose a wheat 3D plot segmentation dataset, quantify the plot canopy height via combination with PointNet++, and generate 4 nitrogen utilization-related vegetation indices via index calculations. Six height-related and 24 vegetation-index-related dynamic digital phenotypes were extracted from the digital phenotypes collected at different time points and fitted to generate dynamic curves. We applied height-derived dynamic numerical phenotypes to genome-wide association studies of 160 wheat cultivars (660,000 single-nucleotide polymorphisms) and found that we were able to locate reliable loci associated with height and NUE, some of which were consistent with published studies. Finally, dynamic phenotypes derived from plant indices can also be applied to genome-wide association studies and ultimately locate NUE- and growth-related loci. In conclusion, we believe that our work demonstrates valuable advances in 3D digital dynamic phenotyping for locating genes for NUE in wheat and provides breeders with accurate phenotypic data for the selection and breeding of nitrogen-efficient wheat varieties.

## Introduction

Wheat (*Triticum aestivum*) is one of the most widely distributed, extensively cultivated and highest-yielding cereal crops in the world [1–3]. Over the past 50 years, with the continuous development of agricultural production, global food production has shown a general upward trend with periodic fluctuations [4]. In this process, the introduction of dwarfing genes and the widespread use of nitrogen fertilizers play pivotal roles in sustaining the increasing yield of wheat [5,6]. However, as food production has increased, so have the costs of agricultural production. As a result, improving crop nitrogen use efficiency (NUE) has become a critical means of achieving agricultural sustainability [6]. A 1% increase in crop NUE has the potential to save up to US$1.1 billion annually in global agricultural production costs [7]. Nitrogen is an indispensable essential nutrient for the growth and development of wheat, making the breeding of nitrogen-efficient wheat varieties a key way to promote agricultural sustainability [8,9]. However, owing to the plethora of genetic network variables controlling NUE, finding and identifying key NUE-related genes and elucidating their associated roles has long been a scientific challenge [10].

With the continuous advancement of genomics and sequencing technologies, genome-wide association studies (GWASs) have been widely applied to several crops, such as rice, maize, wheat, barley, oilseed rape, and soybean, allowing the precise mapping of quantitative trait loci (QTLs) and the identification of key candidate genes associated with complex quantitative traits [11,12]. These studies cover a wide range of traits, including yield, agronomic traits, quality, and disease resistance [13–15]. In recent years, the sequencing of the wheat genome and advances in biotechnology have greatly improved our understanding of the genetic basis and molecular mechanisms of wheat [16]. This indicates the beginning of a critical phase in wheat functional genomics [17]. NUE, a complex quantitative trait, is subject to coordinated regulation by multiple genes [18]. Different wheat genotypes present notable genetic and physiological differences in NUE [19]. Therefore, a comprehensive study of the physiological, biochemical, and genetic variation among different genotypes is crucial for breeding wheat varieties with high NUE. For example, in one study, a genome-wide association analysis was performed on 214 wheat cultivars under 8 different environmental conditions, and 28 NUE-related traits were examined. This research successfully identified several candidate genes, including NADH-GOGAT, which is associated with nitrogen uptake; glutamine synthetase (GS1), which is associated with nitrogen recycling efficiency and straw nitrogen concentration; and photoperiod sensitivity (PpdD1), which is associated with the harvest index and flowering stage nitrogen concentration [20]. Three hundred eighty-nine wheat accessions were genotyped via the Wheat 660K single-nucleotide polymorphism (SNP) array, and 8 NUE-associated agronomic traits were subjected to GWAS, which identified 347 QTLs, including 11 stable QTLs [21]. These findings identify important candidate genes for future breeding efforts to improve NUE in wheat varieties. This highlights the important contribution of genome-wide association analysis in elucidating the genetic mechanisms underlying complex traits, with the potential to drive genetic improvement and sustainable agricultural development in cereal crops.

However, large-scale, high-throughput, field-based analysis of nitrogen use phenotypes remains a technical bottleneck, limiting our ability to dissect the genetic basis of quantitative traits [22]. In recent years, the development of computer vision technology and deep learning algorithms, combined with high-throughput plant phenotyping platforms, has opened many new research avenues and application scenarios for plant research, breeding, and agricultural production [23]. Unmanned aerial vehicle (UAV)-based plant phenotyping has undergone rapid development over the past decade [24]. UAV remote sensing technology, which is equipped with high-resolution cameras or multispectral sensors, can capture high-resolution images of field crops and provide a rich set of phenotypic characteristics related to wheat yield. These characteristics include plant growth status, leaf color, and vegetation cover [25]. In addition, by combining image processing and deep learning algorithms, analytical operations such as feature extraction and object detection and classification can be performed on wheat images acquired by UAVs, and an automated extraction and quantitative analysis process for wheat yield phenotype characteristics can be constructed [26]. The growth process of wheat can be tracked and monitored through regular UAV aerial photography, and timely information on changes in wheat yield phenotypes can be obtained [27]. With the decreasing cost of UAVs and image sensors, improvements in flight control software, and the development of powerful UAV analysis software, many research teams have integrated UAV phenotyping into field breeding and cultivation studies [28]. For example, to study yield-related traits, high-resolution visible (e.g., RGB) cameras, multispectral and hyperspectral devices, light detection and ranging, and thermal infrared sensors are widely used for UAV phenotypic data collection to analyze yield-related phenotypic traits on the basis of the detection of plant morphological and spectral characteristics [29,30].

Multiscale phenotypic data collection and associated analysis methods using UAVs are currently being applied in crop research [31,32]. Researchers have used the morphological, spectral, and textural characteristics of crops at different scales to measure traits related to yield, stress resistance, and reproductive processes. However, capturing static phenotypic traits of wheat at specific points in time via UAVs often overlooks the 3-dimensional (3D) morphological changes that occur during wheat growth [33]. Key phenotypic traits, such as height, vary over time and space. Therefore, continuous monitoring of phenotypic changes in wheat at key growth stages via UAVs is necessary to capture long-term phenotypic variation and intervarietal differences [34]. Consequently, as multiscale phenotype acquisition technology and analysis methods advance, the combination of long-term phenotype analysis approaches will be highly valuable to wheat breeders and researchers. To address these challenges, we developed an automated pipeline for multiscale phenotypic analysis of wheat in the field via data obtained from UAV-based 2-dimensional (2D) orthomosaic images (orthomosaic images are remote sensing images with orthographic projection properties) and 3D point clouds (point clouds are a set of data points in space that include 3D coordinates [*X*, *Y*, and *Z*], colors, and classification values) in wheat experiments. The main contributions of this study are as follows:

• We collected phenotypic data of field wheat at critical growth stages under different nitrogen gradients via UAVs and established datasets of 3D point cloud segmentation and 2D canopy layer images for wheat plots. We introduced a high-throughput process for extracting point clouds from field wheat plots and quantifying 3D plot phenotypic traits through deep-learning-based point cloud segmentation. We also extracted multispectral and texture features from the 2D canopy layer image dataset via spectral analysis algorithms.
• On the basis of the acquired multiscale digitized phenotypic data of wheat at different growth and development stages (3D plots and 2D canopy layers), we constructed a dynamic collection of phenotypic traits covering the entire wheat growth cycle. We analyzed phenotypic changes under different nitrogen gradients during wheat development and digital phenotypic differences between wheat varieties in terms of NUE. These findings validate the applicability of multiscale digital phenotyping in field wheat breeding research.
• Finally, to validate the utility of this pipeline in research, we integrated phenotypic traits with GWAS, applying dynamic traits derived from static traits to genetic map investigations and identifying candidate genes.

This approach offers a promising solution for advancing our understanding of wheat genetics, improving breeding efforts and facilitating more efficient crop production.

## Materials and Methods

### Wheat germplasm, field trials, and multiscale phenotyping data generation

**Fig. 1.**
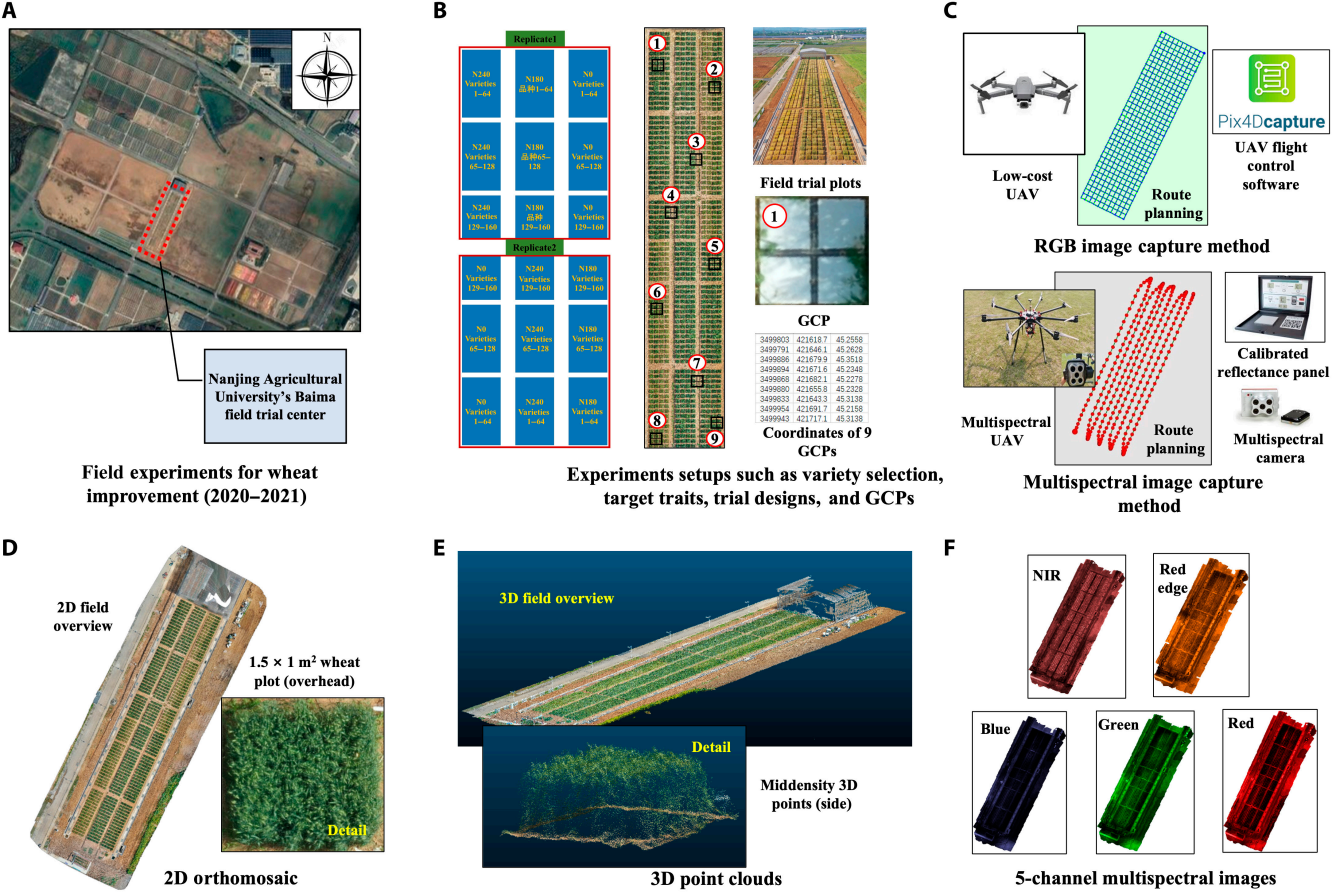
Development of a comprehensive procedural framework for the acquisition and preprocessing of phenotypic data via unmanned aerial vehicles (UAVs). This includes the systematic acquisition of 2-dimensional (2D) RGB and multispectral imagery, followed by the subsequent processes of 2D image stitching and the generation of high-resolution 3-dimensional (3D) point clouds, providing detailed plant-level resolution for the trial field. (A and B) The trial center served as the geographical epicenter for an extensive survey of 160 different wheat varieties. This process involved intricate planning of experimental treatments, experimental layouts, and field setups, including the strategic positioning of ground control points (GCPs). (C) Drones and equipped sensors, as well as flight paths. (D and E) Data preprocessing was systematically performed to generate high-resolution 2D orthomosaics and detailed 3D point clouds, providing plot-level plant resolution for the experimental field. (F) Stitched 5-channel multispectral image.

During the 2020 to 2021 wheat growing season in Nanjing (31°36′57.8″N, 119°10′46.1″E; Fig. 1A), we conducted a phenotypic analysis experiment on 160 naturally occurring wheat populations collected from the middle and lower reaches of the Yangtze River and the Huang–Huai–Hai Plain regions. The experiment included different nitrogen gradient treatments (Fig. 1B) with 2 replicates and 3 nitrogen levels (0, 180, and 270 kg N ha^−1^; i.e., N0, N180, and N240, respectively). These wheat varieties were sown in 1.5 × 1 m plots, with 20-cm spacing, 5 rows per plot, and approximately 450 plants per plot. The base fertilizer, comprising 60% of the total nitrogen (urea), phosphorus (P_2_O_5_), and potassium (K_2_O) fertilizer, was applied at 0 d after sowing (DAS). This was followed by the application of 40% nitrogen fertilizer at 125 DAS, which coincided with the majority of the varieties entering the jointing stage. The crops were cultivated in accordance with standard husbandry and agronomic practices tailored to specific local conditions.

We designed 2 mission plans: (a) We used a DJI Mavic 2 Pro drone (equipped with a high-definition RGB camera, with a maximum image resolution of 5,472 × 3,648 pixels; DJI, Shenzhen, China) to capture RGB images of the wheat field plots at an altitude of 10 m (Fig. 1C, top) and subsequently generated orthomosaic images (Fig. 1D) and 3D point clouds (Fig. 1E) via PIX4Dmapper (version 1.4, Pix4D, Lausanne, Switzerland). (b) We also used a DJI Matrice 600 drone (DJI, Shenzhen, China) equipped with a MicaSense multispectral camera (equipped with a 5-band lens, with a maximum image resolution of 2,464 × 2,056 pixels) to collect multispectral images of wheat field plots at an altitude of 25 m (Fig. 1C, bottom).

These images were processed via PIX4Dmapper to produce orthomosaic images in different spectral bands (Fig. 1F). For the RGB image data collected at different growth and development stages of wheat during 10 sessions in the 2020 to 2021 season, we generated 12 sets of 2D orthomosaic images and 12 sets of 3D point cloud data. For the orthophoto (RGB and multispectral) stitching methods, we used the image-based method for generating 3D point clouds in reference to previous studies [35]. For the multispectral imagery data, preprocessing involved the generation of orthomosaic images, with key steps including camera alignment and georeferencing, resulting in the generation of orthomosaic images in 5 spectral bands. A total of 60 multispectral images were obtained by processing data collected at different growth and development stages of wheat during the 2020 to 2021 season. To avoid unnecessary aerial phenotyping, we performed only 12 flights in 8 growth stages during the season, from seedling to late grain filling, to provide sufficient plant growth and development phenotyping data for phenotypic analysis (seeding, tillering, jointing, booting, heading, flowing, filling, and ripening). We collected data twice at the booting, heading, flowing, and filling stages, so a total of 12 flights were made using 2 types of drones.

### Automated high-throughput analysis pipeline for 3D plot phenotyping of wheat

We developed a comprehensive annotation workflow for field wheat 3D plot point clouds. This process involves the annotation of wheat plot point clouds and non-wheat point clouds, resulting in the creation of a field wheat 3D plot segmentation (W3DPS) dataset. The target plots were loaded into the CloudCompare software, and the ground point clouds were initially filtered via curvature-based and statistically based filtering algorithms [36]. This was combined with manual removal of impurity point clouds (weeds or signs), resulting in distinct point clouds for different wheat varieties. The filtered plot point clouds were labeled “wheat_point_cloud”, whereas the ground and impurity point clouds were uniformly labeled “ground_point_cloud”.

To date, there is no existing automated algorithm specifically designed for 3D plot segmentation of field wheat. Considering the increasing popularity of deep learning techniques in plant research, we employed the 3D semantic segmentation network PointNet++ to train our dataset [37]. PointNet++ is an advanced deep learning architecture designed to work with 3D point cloud data. It builds upon the success of PointNet, improving its performance by addressing some of the limitations inherent in processing unstructured point clouds. The key enhancement introduced in PointNet++ is hierarchical feature learning via a set of nested spatial regions, which allows for better capture of local structures within the point cloud while maintaining the global context.

The model achieves this feature learning through a series of set abstraction layers that progressively reduce the number of points and increase the receptive field, thus capturing multiscale features. This hierarchical approach enables the network to learn from both the local details and the global structure of the input point cloud, making it more effective at tasks such as 3D object classification and segmentation.

**Fig. 2.**
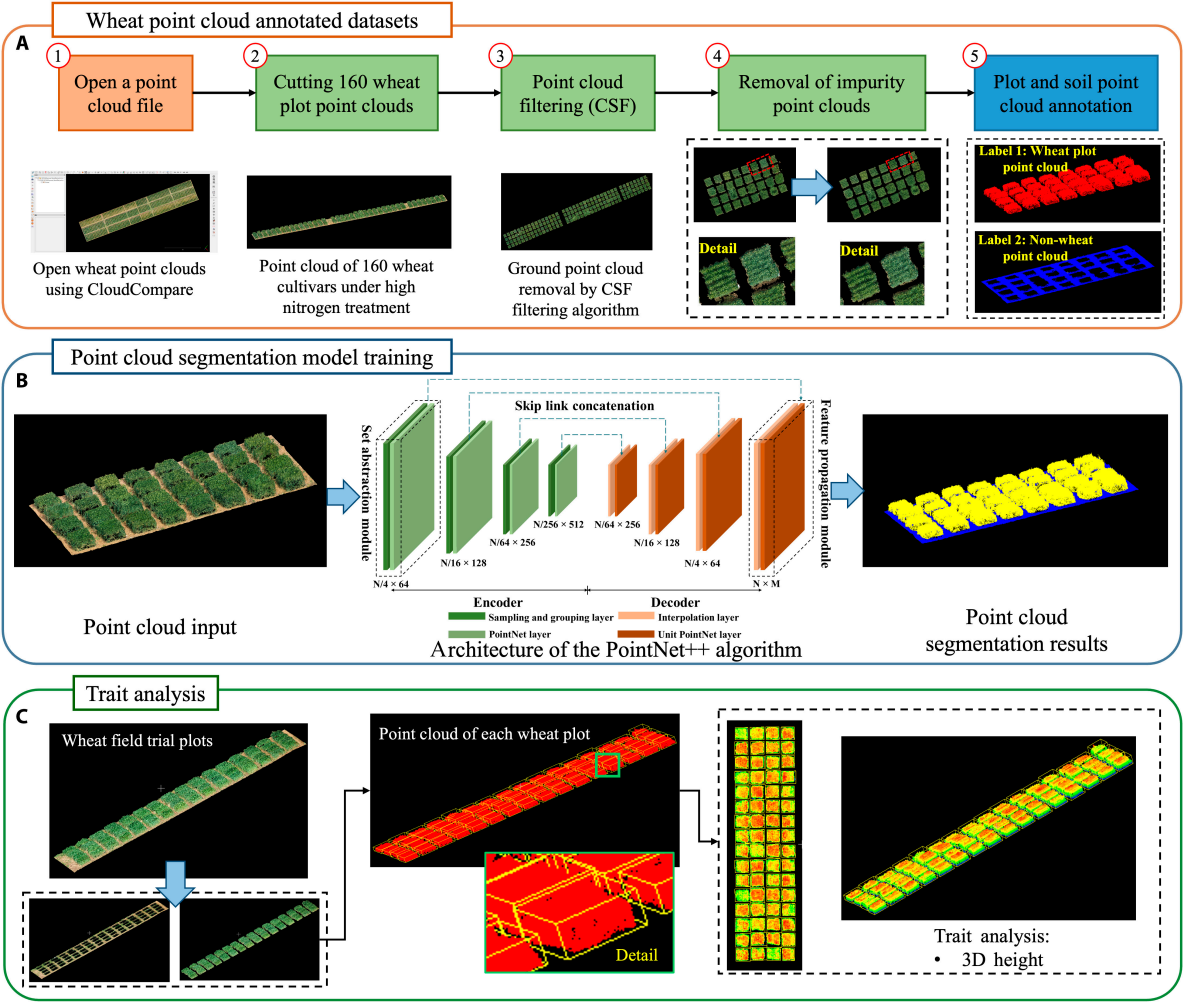
Algorithm steps for processing the 3D point cloud data generated from UAV imagery, including the use of a deep learning 3D point cloud segmentation algorithm to extract different wheat varieties and their 3D plot point clouds combined with a point cloud analysis algorithm to calculate the digitized phenotypic features of each plot point cloud. (A) Combining soil filtering algorithms with manual annotation, the process involves separating soil point clouds from aboveground wheat point clouds. In addition, ground point clouds and plot-specific point clouds are classified and annotated by manually removing impurity point clouds that are unrelated to the ground or wheat. This methodology facilitates the construction of a 3D plot segmentation dataset for field-based wheat analysis. (B) To train the 3D plot segmentation model for field wheat, the Point++ deep learning point cloud segmentation algorithm is used to train the dataset. Upon completion of the segmentation, different categories of point clouds are colored, and .las files are generated for each category. (C) When the trained Point++ model is used to segment point clouds under different nitrogen treatments, processing is limited to 3D point clouds of aboveground wheat plots. For each segmented plot, the outermost enclosing points are calculated, and bounding boxes are drawn, allowing phenotypic traits to be extracted for individual plot point clouds. Using the geographic coordinates on the ground plane, the height of each point within the canopy of the wheat plots is calculated for each bounding box. The average height is then calculated to obtain the 3D height of the plot. CSF, Cloth Simulation Filter.

This model successfully segmented 3D plot point clouds from 12 field experiments under different nitrogen application conditions, producing segmented 3D plot point clouds of field wheat (Fig. 2B) for each nitrogen application scenario. By applying the trained model to 3D point clouds generated at different time points, we segmented the aboveground 3D point clouds of soft wheat. For each segmented point cloud, we performed 3D feature extraction for each plot (Fig. 2C).

### Multispectral phenotyping

We selected 4 vegetation indices (VIs) to assess NUE and growth in wheat. The main reason for selecting these indices was to identify indices used to estimate the canopy N concentration (N%) and content (g·N·m^−2^) that are robust across crop locations and growing conditions. The wavelengths used to derive the indices, formulas, and references are given in Table 1.

**Table 1.** Vegetation indices used in this study

Index	Full name	Formulation	Reference
CCCI^a^	Canopy chlorophyll content index	(NDRE − NDRE_min_)/(NDRE_max_ − NDRE_min)_	[61]
MSAVI	Modified soil-adjusted vegetation index	(2 ∗ *R*_NIR_ + 1 − sqrt((2 ∗ *R*_NIR_ + 1)^2^ − 8 ∗ (*R*_NIR_ − *R*_red_)))/2	[62]
RVI_1	Relative vegetation index 1	*R*_NIR_/*R*_red_	[63]
RVI_2	Relative vegetation index 2	*R*_NIR_/*R*_green_	[64]

NDRE, normalized difference red edge

^a^ CCCI is calculated from the following parameters: NDRE = (*R*_NIR_ − *R*_red_)/(*R*_NIR_ + *R*_red_), NDRE_max_ = 0.61, and NDRE_min_ = 0.24.

### Genome-wide association study

The GWAS was performed using the FarmCPU model with the rMVP package in R [38]. The computationally powerful statistical approaches built into rMVP, including efficient mixed-model associations and factored spectrally transformed linear mixed models, were selected to optimize the computational speed and statistical performance. The effects of relatedness and population structure of the first 3 principal components were also calculated by rMVP as covariates to reduce false positives. Markers with a −log_10_(*P* value) ≥ 3.5 were considered a significant SNPs for GWAS results. Manhattan plots and quantile–quantile plots were automatically generated via rMVP to visualize the significant markers and *P* value distributions. Candidate genes associated with each significant marker were searched and annotated via the marker information module in the WheatOmics and Dr. Tom databases (https://biosys.bgi.com), respectively.

### DNA extraction and genotyping

Genomic DNA extraction was performed via the cetyltrimethylammonium bromide method [39]. All the cultivars tested were genotyped at the China Golden Marker facility in Beijing, China. Genotyping was performed via the commercially available Wheat660 SNP array from Santa Clara, United States, which contains 660,000 polymorphic SNP markers. The physical positions of these SNP markers were accurately mapped against International Wheat Genome Sequencing Consortium RefSeq v1.0, available at http://www.wheatgenome.org. Rigorous SNP quality control procedures were performed via Axiom Analysis Suite v5.2, developed by Thermo Fisher Scientific and based in Massachusetts, United States. SNPs with more than 10% missing data points and minor allele frequencies below 5% were systematically removed through this quality control pipeline. After filtering, 409,976 high-quality SNPs remained available for the GWAS.

## Results

### High-speed dynamic digital phenotypic generation

In plant research, dynamic phenotyping not only provides intermediate phenotypes of target traits (e.g., by fitting change curves) but also provides detailed parameters of phenotypic changes in target traits (e.g., the growth rate of plant height during the nitrogen response), i.e., measuring phenotypic changes in traits over time, which provides the basis for analyses of genetic variation [40]. Indeed, spatial and temporal phenotypic variation, which can link plant responses to external stimuli, such as gene expression and regulatory factors, increases the ability to detect loci of interest [41]. Many external factors (e.g., soil type, climatic conditions, growth stage, and N availability) are associated with N-responsive gene expression, which changes as the reproductive period progresses, so dynamic phenotypic changes allow the dynamic resolution of key processes of nitrogen uptake, utilization, and translocation in plants. This will also highlight the need for and feasibility of selecting key N-responsive traits at key growth stages and conducting GWAS across key response stages [42].

**Fig. 3.**
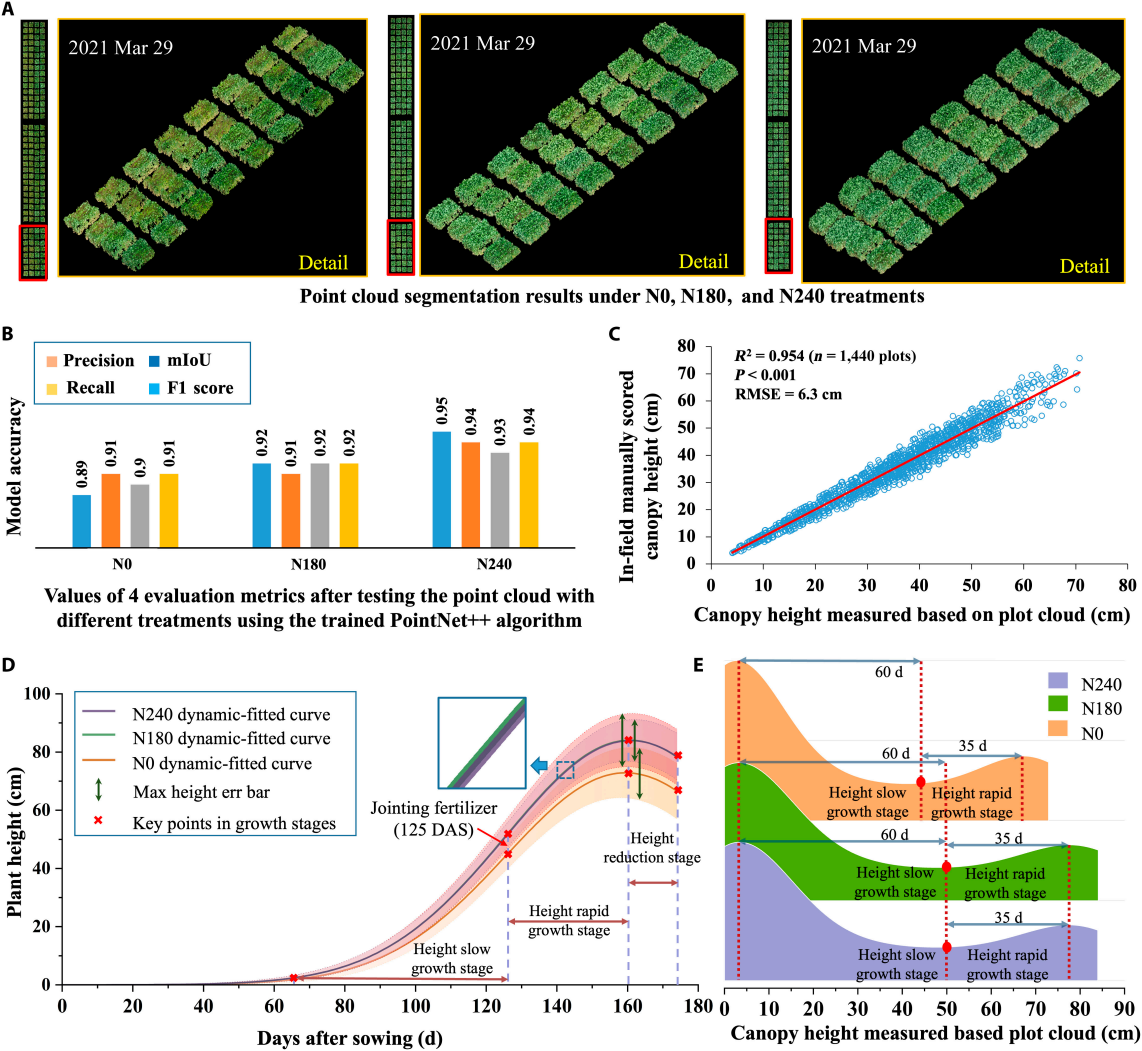
(A) We utilized the PointNet++ model to segment 3D point clouds of wheat plots under 3 nitrogen treatments. This segmentation was conducted on 2021 March 29, highlighting the effectiveness of the constructed wheat plot point cloud processing pipeline in real-field applications. (B and C) The performance of the trained PointNet++ model was rigorously evaluated via 4 model metrics. The results indicated consistently high scores, exceeding 90 for all 4 metrics across the different nitrogen treatments, confirming the model’s robustness. We also correlated the canopy heights of 1,440 wheat plots with those of manual statistics (*R*^2^ = 0.954, *P* < 0.001, root mean square error [RMSE] = 6.3 cm/plot). (D and E) Based on 10 wheat heights, we plotted fitted curves to analyze height changes and error intervals under different nitrogen treatments and analyzed different stages of height changes during the height change process to extract dynamic digitized phenotypic traits. DAS, days after sowing; mIoU, mean intersection over union.

We observed variations in the color, texture, and point cloud density of wheat under different nitrogen treatments, and different wheat varieties presented different growth patterns. Analyzing NUE between different wheat varieties on the basis of a single set of static data has proven challenging. Therefore, inspired by previous research, we delved into the analysis of dynamic wheat phenotypes. Dynamic phenotypes reflect different trait changes and variations over time as plants grow and develop. Through 3D plot segmentation and trait analysis, we obtained a time-series-based set of 3D canopy heights for the entire wheat growth cycle. For the key phenotypic trait of 3D canopy height, which reflects wheat growth and development, we generated phenotype dynamic change curves by fitting to analyze the response of field wheat to nitrogen uptake and use efficiency under different nitrogen conditions. We used height data for 160 local wheat cultivars collected 10 times between sowing and maturity, with relatively uniform time intervals. During the grain filling stage, the wheat canopy height tends to decrease. Inspired by phenotyping research in rice [35], we used Gaussian functions to approximate the data points for each collection and plotted 3 mean-fitted curves for canopy height under the 3 nitrogen treatments: $f{\left(x\right)}_{\text{height}-N0}$, $f{\left(x\right)}_{\text{height}-N180}$, and $f{\left(x\right)}_{\text{height}-N240}$. We calculated the derivatives of the fitted curves for wheat height changes. Under the N180 and N240 treatments, the wheat height changes and height change rates were almost identical. However, in the N0 treatment, both the height change rates and height change rates were relatively lower than those in the N180 and N240 treatments from 60 to 125 DAS (height slow growth stage). After heading (125 DAS), the height changes and rates were greater in the N180 and N240 treatments than in the N0 treatment (125 to 160 DAS, rapid height growth stage). Under all 3 nitrogen treatments, the wheat height reached its maximum at 160 DAS. However, the height maximum under N240 and N180 was 15 cm greater than that under N0, and the error intervals were more concentrated for N240, indicating fewer extreme height cases than for N0. N0 had the widest range of error intervals, indicating that extreme heights were more likely to occur under nitrogen deficiency. After the grain filling stage, the decrease in the mean canopy height was similar for all the N treatments, with a decrease of approximately 5 cm. We also calculated segmentation performance metrics (precision, recall, mean intersection over union, and F1 score) for different nitrogen application conditions. Correlation analysis between the model’s average 3D point height for 1,440 plots and manually measured heights revealed strong correlations, indicating the model’s effectiveness in 3D plot segmentation of field wheat suitable for practical field environments (Fig. 3B).

We also calculated the changes in wheat growth at different heights under the 3 treatments (Fig. 3E). During the 65-d period of the slow height growth stage, before tillering fertilizer application, the mean wheat height in the N240 and N180 treatments was approximately 50 cm, whereas in the N0 treatment, it was approximately 42 cm. After tillering fertilizer application in the rapid height growth phase, the changes in wheat height were faster in N240 and N180 than in N0. On the basis of the analysis of height changes in wheat under different nitrogen treatments, we calculated a number of dynamic height-related phenotypes (Table 2).

**Table 2.** Six dynamic digital phenotypic traits derived from static height traits. Their equations, normalization, references, and biological relevance are provided in Note S1.

Signal	Dynamic traits	Measurement unit
Morphological traits (plot-based static traits for all growth stages of wheat)	1. Maximum canopy height days (*height_md_*)	Days
	2. Maximum canopy height (*height_max_*)	cm
	3. Start data of rapid growth (*height_srg_*)	DAS
	4. End data of rapid growth (*height_erg_*)	DAS
	5. Rapid growth days (*height_drg_*)	Days
	6. Height growth rate (*height_gr_*)	None

### GWAS using height-related digital dynamic phenotypic traits

**Fig. 4.**
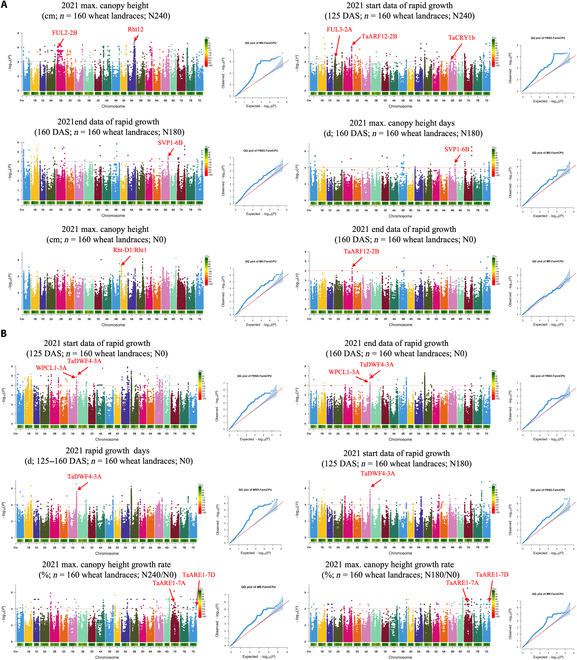
Manhattan plots and quantile–quantile (QQ) plots for dynamic digital height phenotypic traits subjected to a genome-wide association study (GWAS) for 160 wheat landraces in 2021. The significance threshold is shown by the horizontal orange dashed line. Known genes that colocalized with identified loci are indicated by red arrows. Known genes that colocalized with identified loci are indicated by red arrows. (A) The ${\text{height}}_{\max}$ trait was used to identify 2 single-nucleotide polymorphisms (SNPs) colocalized with known height-related genes: FUL2-2B (chromosome 2B) and Rht12 (chromosome 5A) under the N240 treatment [53]. The ${\text{height}}_{\mathrm{srg}}$ trait was used for the identification of 3 SNPs colocalized with known height-related genes: FUL3-2A (chromosome 2A), TaARF12-2B (chromosome 2B), and TaCRY-1b (chromosome 6B) [54]; under the N180 treatment, both ${\text{height}}_{\mathrm{erg}}$ and ${\text{height}}_{\mathrm{md}}$ were used for the identification of 1 SNP colocalized with a known height-related gene: SVP1-6B (chromosome 6B) [55]; under the N0 treatment, ${\text{height}}_{\max}$ and ${\text{height}}_{\mathrm{erg}}$, respectively, were used to identify 2 SNPs colocalized with known height-related genes: Rht-D1 (${\text{height}}_{\max}$, chromosome 4D) and TaARF12-2B (${\text{height}}_{\mathrm{erg}}$, chromosome 2B). (B) Two signals identified under the N0 treatment, using both ${\text{height}}_{\mathrm{srg}}$ and ${\text{height}}_{\mathrm{erg}}$, were identified close to the WPCL1-3A gene and the TaDWF4-3A gene on chromosome 3A, which is highly correlated with nitrogen use efficiency (NUE), and in addition, one signal identified by ${\text{height}}_{\mathrm{drg}}$ was also on chromosome 3A close to the NUE-associated TaDWF4-3A gene; under the N180 treatment, the signal identified by the ${\text{height}}_{\mathrm{srg}}$ trait was close to the NUE-related TaDWF4-3A gene on chromosome 3A; and under the N240/N0 and N180/N0 ratio calculations, the 2 signals identified using the ${\text{height}}_{\mathrm{gr}}$ trait were close to the NUE-related TaARE1-7A gene on chromosome 7A and the TaARE1-7A and TaARE1-7D genes on chromosome 7D [56,57].

We used 6 height-related dynamic digital phenotypic traits in a GWAS analysis of 160 wheat landraces. We identified several significant SNPs associated with height-related traits (from the 2020 to 2021 season) and presented them in Manhattan plots and quantile–quantile plots, with a gray dotted line indicating the threshold of *P* value and a false detection rate of 0.2. In total, we identified 81 SNPs associated with height and 72 SNPs associated with NUE. For example, when the ${\text{height}}_{\max}$ (maximum canopy height) trait was used, the signal on chromosome 5A (−log_10_(*P*) = 6.32, indicated with a red arrow; Fig. 4A, upper left) was c. 272 kb from the Rht12 gene [43], which is known to have mono effects on plant height. On chromosome 2B, using the ${\text{height}}_{\mathrm{srg}}$ trait, the signal (−log_10_(*P*) = 6.21, indicated with a red arrow; Fig. 4A, upper right) identified was c. 50.2 kb from the TaARF12-2B gene, which is known for its pleiotropic effects on plant architecture, spike length, speed per spike, and grain weight [44].

We found that 6 phenotypes and genome-wide association analyses were also able to locate significant genetic loci associated with NUE. For example, when the ${\text{height}}_{\mathrm{srg}}$ and ${\text{height}}_{\mathrm{erg}}$ traits were used, the signals on chromosome 3A (−log_10_(*P*) = 5.44 and −log_10_(*P*) = 5.14, indicated with red arrows; Fig. 4B, upper) were c. 109 kb and c. 189 kb from the WPCL1-3A gene and the TaDWF4-3A gene [45,46], respectively, which are known for their NUE and wheat productivity. The SNPs identified above demonstrate that the dynamic numerical height phenotypes we used can be directly used for gene mining related to wheat height and NUE. We used the same method of calculating dynamic phenotypes for the extraction of dynamic phenotypes for the 4 VIs.

### VI dynamic phenotypic generation

**Fig. 5.**
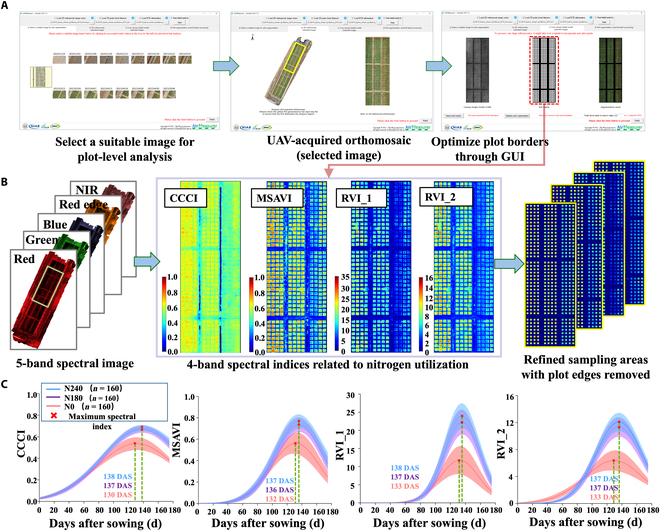
Plot masks of UAV RGB images and multispectral images are combined for the dynamic phenotype extraction of vegetation indices (VIs) on the basis of multispectral images. (A) The RGB image mask generation function of AirMeasurer was used to obtain the plot canopy masks of 160 wheat varieties. (B) On the basis of the existing 5-channel stitched spectral images combined with the formula of the VI and the spectral fusion images of the 4 VIs, the mask overlay on the VI image was segmented, and the static VIs of the plot canopy with the removal of marginal effects were obtained. (C) Based on the wheat spectral images of 10 flights, we plotted fitted curves to analyze the changes and error intervals of the 4 VIs under 3 nitrogen treatments and analyzed the different rates of change during the changes in the VIs to extract the dynamic phenotypic traits on the basis of the VIs. NIR, near infrared; GUI, graphical user interface.

We extracted the phenotypes via the dynamic change curves of the 4 VIs. Combined with the excellent performance of the dynamic height phenotypes in locating wheat height and NUE genes, the dynamic phenotypes derived from the fitted curves of the VIs can also be used to mine and locate NUE genes [47]. For the data processing of single UAV images, we first used the AirMeasurer software to generate the cell segmentation mask of RGB images (Fig. 5A); according to the previous preprocessing of multispectral images, we obtained the multispectral images of the test plot in 5 bands, and through the fusion of the images of the different bands, we produced the reflectance images of the 4 VIs and superimposed the generated masks on the 4 reflectance images. A total of 4 VIs of 160 species were produced as static data (Fig. 5B). On the basis of the methodology for calculating highly dynamic numerical phenotypes (Fig. 5C), fitted curves for the VIs were simultaneously generated, and 6 dynamic phenotypic traits for the 4 VIs were extracted (Table 3).

**Table 3.** Six dynamic digital phenotypic traits derived from static VIs. Their equations, normalization, references, and biological relevance are provided in Note S2.

Signal	Dynamic traits
Spectral traits (plot-based static traits, all growth stages of wheat)	1. VIs of maximum canopy days (CCCI_md_, MSAVI_md_, RVI_1_md_, and RVI_2_md_)
	2. Maximum VIs (CCCI_max_, MSAVI_max_, RVI_1_max_, and RVI_2_max_)
	3. Start data of VIs of rapid change (CCCI_src_, MSAVI_src_, RVI_1_src_, and RVI_2_src_)
	4. End data of VIs of rapid change (CCCI_erc_, MSAVI_erc_, RVI_1_erc_, and RVI_2_erc_)
	5. VIs of rapid change days (CCCI_drg_, MSAVI_drg_, RVI_1_drg_, and RVI_2_drg_)
	6. VIs of rapid change rate (CCCI_rcr_, MSAVI_rcr_, RVI_1_rcr_, and RVI_2_rcr_)

### The GWAS results support the validity of the dynamic phenotypes generated by VIs in the genetic resolution of NUE

**Fig. 6.**
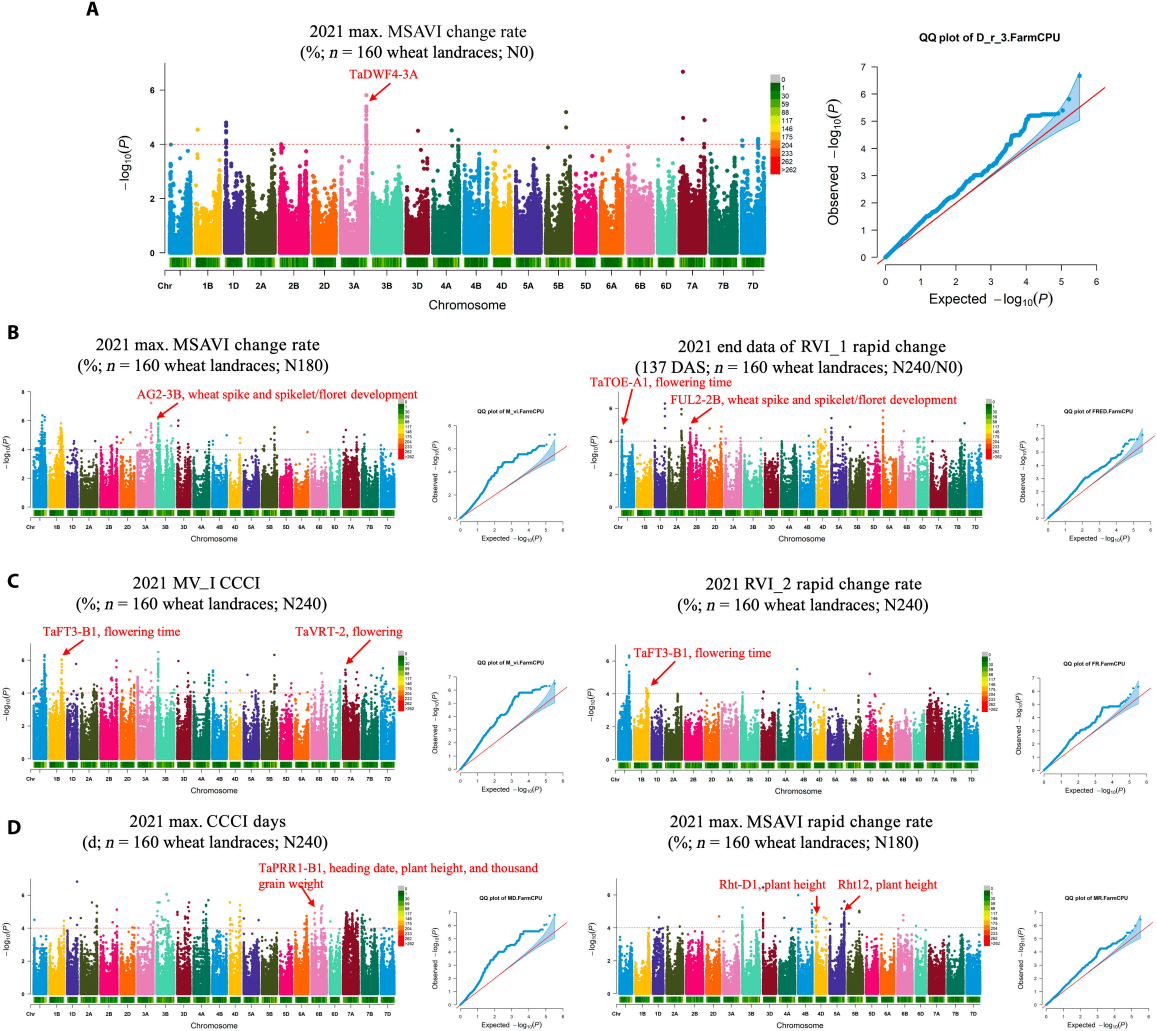
Manhattan plots and QQ plots for VI dynamic phenotypic traits subjected to a GWAS for 160 wheat landraces conducted in 2021. The significance threshold is shown by the horizontal orange dashed line. Known genes that colocalized with significant loci are indicated by red arrows. Known genes that colocalized with significant loci are indicated by red arrows. (A) Phenotype ${\text{msavi}}_{\mathrm{mcr}}$ was generated via MSAVI under N0 treatment for the identification of one identified SNP colocated with a known NUE-related gene, TaDWF4-3A (chromosome 3A), which was also localized when the dynamic digital height-related phenotype GWAS was used. The GWAS was also localized. (B) Use of ${\text{msavi}}_{\mathrm{rcr}}$ and $\mathrm{rvi}1_{\mathrm{erc}}$ for 3 identified SNPs colocalized with known wheat growth-related genes: AG2-3B (chromosome 3B), TaTOE-A1 (chromosome 1A), and FUL2-2B (chromosome 2B), with FUL2-2B also localized when the dynamic digital-height-related phenotype was used. (C) The dynamic phenotypes ${\text{ccci}}_{\max}$ and $\mathrm{rvi}1_{\mathrm{rcr}}$, generated via CCCI and RVI_2, were both able to identify one SNP, TaFT3-B1 (chromosome 1B), which colocalized with genes known to be associated with flowering time [58], in addition to ${\text{ccci}}_{\max}$, which was able to identify an additional identified SNP, TaVRT-2, which was associated with flowering, under N240 treatment. (D) Use of ${\text{ccci}}_{\mathrm{md}}$ and ${\text{msavi}}_{\mathrm{rcr}}$ for 3 SNPs colocalized with genes known to be highly associated with wheat: TaPRR1-B1 (chromosome 6B), Rht-D1 (chromosome 4D) [59], and Rht12 (chromosome 5A). The above GWAS analyses revealed that the dynamic phenotypes derived from NUE-related spectral indices were able to localize candidate loci, and we did not use any manually scored traits for the GWAS. In doing so, we aimed to validate that the digital phenotypes can replace the manually scored phenotypic data to a certain extent and assist in more precise SNP localization. Therefore, further applications of digital phenotyping in genetic breeding will be further elaborated in our discussion [60].

To further assess the value of digitized NUE traits in accurately quantifying the dynamic field nitrogen response, we selected data from each of the static points of canopy chlorophyll content index, modified soil-adjusted vegetation index, relative vegetation index 1, and relative vegetation index 2 to generate fitted curves, and dynamic phenotypes were generated for the GWAS. A total of 298 SNPs were ultimately identified as associated with growth and development, with a total of 9 loci associated with NUE (Fig. 6).

## Discussion

### W3DPS: An open dataset for 3D plot segmentation of field wheat

With the availability of increasingly more open-source image-based deep learning datasets, the development and high quality of image training datasets are no longer bottlenecks for computer vision and plant research communities in realizing the full potential of artificial intelligence. Researchers and breeders are now also focusing on the 3D morphology and spatial structure of plants; thus, researchers need to further facilitate the quantification of 3D plant traits through the use of 3D point clouds [48,49]. At present, few plant 3D point cloud datasets exist, so we created the W3DPS open dataset to facilitate the segmentation of wheat field plots [50]. W3DPS contains ground point clouds and wheat plot point clouds labeled from 10 point cloud datasets of wheat field trial plots, with a total of 9,600 wheat plots of 160 wheat varieties, covering the whole reproductive period of wheat varieties in the middle and lower reaches of the Yangtze River and the Huang–Huai–Hai Plain. We hope to further expand the dataset by adding point cloud data of different wheat varieties from around the world. We envision that the W3DPS dataset can be widely used to facilitate the development of algorithms for 3D plot segmentation of wheat in the field and to facilitate whole-cycle phenotyping of wheat and related breeding or agricultural production.

### The UAV-based data acquisition method enables high-throughput dynamic phenotyping of wheat in 3D plots in the field

In this study, we used an economical UAV to acquire RGB images of the field, reconstructed a 3D point cloud of the wheat test plot, and segmented the ground point cloud and the wheat plot point cloud via the 3D deep learning segmentation algorithm PointNet++. The point cloud segmentation algorithm used can achieve better segmentation accuracy and efficiency. For the acquired point clouds of the wheat plots in the field at different fertility periods, we obtained static 3D phenotypes, such as 3D height, which is the average of the height values of the plot canopy point clouds, through the traditional processing methods of 3D point clouds. On the basis of the static numerical phenotypes of the heights of different wheat varieties at different fertility periods, we obtained the height change curves of 160 wheat varieties with different nitrogen gradients through Gaussian fitting methods. On the basis of the height change curves, we extracted 6 dynamic numerical phenotypes, including the rate of change in the wheat canopy height and the number of days in the rapid change phase of the canopy height. We believe that the static phenotypes of wheat have been extensively studied by previous researchers and that the dynamic phenotypes can further reflect the dynamic changes in wheat based on long time series. In addition, for the multispectral images acquired by the UAV, we also stitched 5 bands of spectral images and calculated and acquired 4 VIs related to nitrogen use, and on the basis of the calculation method of 3D dynamic numerical phenotypes, we acquired nitrogen-related dynamic numerical phenotypic traits on the basis of VIs.

Phenotyping methods based on UAV-collected images have been shown to replace manual measurements [51], freeing individuals from heavy physical measurements in the field, and the pipeline analysis of UAV-collected images and multiscale phenotypic data generated in this study allows phenotyping and analysis of wheat plots in a process-oriented manner. This cutting-edge technology notably improves the efficiency and accuracy of wheat phenotyping in the field and can be used to collect phenotypes throughout the reproductive period of wheat and support large-scale commercial breeding programs.

### Combining high-throughput, multiscale digital phenotyping and genotyping improves the accuracy, statistical power, and resolution of genetic studies

Dynamic or long-term traits combined with molecular markers can reveal gene expression regulation over time; all phenotypic data in this study used digital phenotypes, and dynamic phenotypes generated from static digital phenotypes were examined to identify relevant loci. We identified consistent gene loci from local wheat varieties with signals related to height, NUE, growth, and flowering associated with plant height, NUE, and growth regulation, demonstrating the value of dynamic phenotyping in the study of genetically diverse local wheat varieties. In addition, the high marker density of the 660K chip we used somewhat compensates for the limitation that the wheat populations we used were not large enough, which is particularly important for wheat because of its large and complex genome size. In this study, we localized several previously unknown strong signals that may be of immense value in identifying the effects of individual allelic differences that collectively contribute to the regulation of trait expression. While the accurate targeting of known genes and the localization of unknown signals demonstrate the reliability of high-throughput dynamic digital phenotyping and genotypic association analysis, precise phenotyping and trait refinement are also important in wheat phenotypic genetics; i.e., the higher the phenotypic precision and the more refined the traits in the time series are, the greater the phenotypic interpretability of the resulting loci.

### Limitations and future developments

Wheat is a crucial staple food in many countries worldwide. To evaluate the NUE of wheat varieties under field conditions and identify the genetic loci regulating NUE, large-scale, multiscale dynamic phenotyping is essential for GWASs, as wheat NUE is influenced by multiple genes [52]. In this study, we present a multiscale digital phenotyping pipeline that integrates full life-cycle phenotyping and leverages artificial intelligence algorithms to quantify wheat phenotypic dynamics. This is achieved using RGB and multispectral images captured by drones, processed through 3D deep learning algorithms and VI computations.

To develop this analysis pipeline, we first created the open W3DPS dataset. This dataset includes 9,600 wheat plot point clouds, derived from 10 data collections of 160 wheat varieties annotated by wheat experts and computer developers, along with ground point clouds. Using the 3D point cloud segmentation algorithm PointNet++, we extracted the point clouds of the wheat plots and obtained 3D digitized phenotypes through point cloud analysis. We then identified 6 dynamic height phenotypes of wheat throughout its reproductive period for GWAS analysis. Based on these height phenotypic traits, we located 9 published genes related to wheat height and 5 genes associated with NUE, validating the use of the PointNet++ algorithm.

Our findings demonstrate the practical applicability of the 3D digital dynamic phenotyping method for NUE gene mining in wheat. Additionally, we utilized the digitized dynamic phenotypes of 4 VIs for GWAS analysis of NUE and successfully localized the TaDWF4-3A gene in both height and spectral phenotypic GWAS analyses. We also identified several wheat growth- and development-related gene loci. Looking ahead, we plan to optimize the 3D point cloud segmentation model and explore new methods for wheat plot segmentation. In this study, we classified the ground and plot point clouds, but we encountered issues with weed point clouds being misclassified. To address this, we will use filtering algorithms to remove weed point clouds and employ grid sorting to obtain the 3D plot point cloud for each species. We aim to directly obtain the 3D point cloud of each plot through 3D point cloud target detection, eliminating the need for point cloud filtering and improving the speed of 3D plot acquisition without compromising detection accuracy. Furthermore, we will refine the dynamic phenotype extraction method. As different wheat varieties have varying fertility processes, making intervarietal comparisons of dynamic phenotypes within the same fertility period challenging, we will divide the reproductive period for each variety and analyze dynamic phenotypes within their critical reproductive periods. This approach will facilitate subsequent gene targeting in GWAS. We hope that our phenotype extraction method and future improvements can be applied to various wheat field trials, including abiotic stress, variety selection, and agronomic trait analysis. Although we have identified loci related to NUE in this study, we plan to further mine new SNPs associated with NUE by using a larger wheat variety population and conducting multisite trials.

## Data Availability

The source code is distributed under the Creative Commons Attribution 4.0 international license, permitting academic use, distribution, reproduction in any medium, provided you give appropriate credit to the original authors and the source, provide a link to the Creative Commons license, and indicate if changes were made. Unless otherwise stated, the Creative Commons Public Domain Dedication (http://creativecommons.org/licenses/by/4.0) waiver applies to the data and results made available in this paper. The source code, testing data, and other datasets supporting the results presented here are available at https://pan.quark.cn/s/afbf9025b19e and https://pan.quark.cn/s/47e91f9d6c9c. Other data and user guides are openly available on request.

## Supplementary Materials

Supplementary 1 Notes S1 and S2
